# A Mixed‐Method Feasibility Study of an Online Group Program for Pregnant Women With ADHD

**DOI:** 10.1111/jep.70755

**Published:** 2026-11-15

**Authors:** Katrine Marcussen, Rikke Kirstine Kristensen, Amalie Thea Jensen, Trine Munk‐Olsen, Anja Elliott Fris, Kathrine Bang Madsen

**Affiliations:** ^1^ Research Unit of Child and Adolescent Psychiatry, Department of Clinical Research University of Southern Denmark Odense Denmark; ^2^ Department of Child and Adolescent Psychiatry Southern Denmark Odense University Hospital, Region of Southern Denmark Odense Denmark; ^3^ Department of Clinical Research, Odense University Hospital Research Unit for Digital Psychiatry University of Southern Denmark Odense Denmark

**Keywords:** ADHD, feasibility study, maternal mental health, online group intervention, postpartum, pregnancy

## Abstract

**Background:**

Pregnant women with ADHD may experience specific challenges related to executive functioning, emotional regulation, and preparation for motherhood. ADHD Mother's Online Resources (AMOR) is a 10‐week online group program developed to provide ADHD‐adapted psychoeducation, practical coping strategies, and peer support during pregnancy. This study evaluated the feasibility and acceptability of AMOR among pregnant women with ADHD in Denmark.

**Methods:**

We conducted a mixed‐methods feasibility study using pre‐ and post‐intervention questionnaires and post‐program individual and focus group interviews. Feasibility outcomes included recruitment, retention, adherence, satisfaction, perceived credibility, and negative effects. Secondary outcomes included self‐reported ADHD symptoms, perceived stress, depressive and anxiety symptoms, emotion regulation difficulties, and ADHD‐related quality of life. Qualitative interviews with 12 participants were analysed using the Framework Method, informed by the Theoretical Framework of Acceptability.

**Results:**

Fifty‐seven women self‐referred within 3 months, and all three groups reached capacity within 24 h. Thirty‐two women were enroled; 27 completed the program (program retention 84.4%) and 25 completed follow‐up questionnaires (follow‐up retention 92.6%), and mean session attendance was 80%. Satisfaction was high with a total mean Client Satisfaction Questionnaire score of 25.76; 88% would recommend the program and 88% intended to continue using the techniques. Reported negative effects were infrequent and mild. Qualitative findings indicated high acceptability, with participants describing the program as relevant, supportive, and meaningful. Perceived benefits included practical strategies, normalization, peer support, increased self‐acceptance, improved partner communication, and greater confidence in communicating with healthcare professionals. Quantitative changes in symptom‐based measures were small, with improvements observed particularly for anxiety symptoms and ADHD‐related quality of life.

**Conclusion:**

Findings suggest substantial unmet need for ADHD‐adapted perinatal support. AMOR appeared feasible, acceptable, and well tolerated among pregnant women with ADHD. Future controlled studies should evaluate its effectiveness and impact on maternal psychosocial outcomes and postpartum well‐being.

**Trial Registration:**

ClinicalTrials.gov identifier: S‐20240074, 11/05/2024.

## 1 Introduction

Attention deficit hyperactivity disorder (ADHD) is a common neurodevelopmental condition that affects both children and adults [1]. For women with ADHD, the reproductive years may present unique challenges. Pregnancy and the transition to motherhood place substantial demands on executive functioning, emotional regulation, organisation and sleep, domains in which many women with ADHD already experience longstanding difficulties [2]. Moreover, coping strategies that have been developed over many years may no longer be effective in the context of pregnancy and caring for a newborn. Together, these factors may increase vulnerability to mental health disorders, and evidence suggests that women with ADHD have an elevated risk of postpartum depression and anxiety [3, 4]. However, even in the absence of clinically significant mental health symptoms, women with ADHD may experience substantial challenges adapting to motherhood [2, 5] affecting daily functioning and confidence in the maternal role [6, 7]. Consequently, support needs among pregnant women with ADHD extend beyond the prevention of mental health disorders and include assistance with coping, self‐management, and adaptation to motherhood.

The primary approach of adult ADHD management is pharmacological treatment, with clinical guidelines typically recommending central stimulant medication in combination with psychological and behavioural treatment [1]. While cognitive behavioural therapy can support individuals with ADHD develop skills in time management, task organization, and impulsivity reduction [8] it has also shown positive results in psychological wellbeing and coping strategies even when delivered online [9, 10, 11]. However, evidence‐based non‐pharmacological treatment options tailored to pregnant and postpartum women with ADHD appear to be limited [12, 13] and pregnant women with ADHD face difficult treatment decisions as guidance on ADHD medication in pregnancy remains limited and is inconsistent across settings [14]. Studies based on Nordic registry data shows that a substantial proportion of women (about 60%) discontinue ADHD medication once pregnancy is confirmed [15]. Such discontinuation may worsen ADHD‐related impairment and increase depressive symptoms [16].

This highlights the need to develop and evaluate scalable, non‐pharmacological perinatal support strategies for women with ADHD during pregnancy and the early postpartum period. One such intervention is ADHD Mother's Online Resources (AMOR), an online group program, developed between 2022 and 2023 by the Danish ADHD Association in collaboration with researchers, clinicians and women with ADHD. The program integrates psychoeducation, cognitive‐behavioural strategies, and peer support and focuses on changing negative thought patterns and behaviours and improve coping with stress and emotional demands.

The AMOR program combines online group‐based meetings over a 10‐week period with a parallel psychoeducational podcast series. The rationale behind the AMOR program is to support women with ADHD in navigating pregnancy and early motherhood by strengthening their confidence in managing the transition to motherhood, social support, and well‐being during pregnancy and early motherhood.

The aim of the present study was to evaluate the feasibility and acceptability of the online group program, AMOR, among pregnant Danish women with ADHD by:

- Assessing recruitment, retention and adherence to the program.
- Evaluating participants' satisfaction, perceived program credibility and potential adverse experiences associated with the program using validated questionnaires.
- Exploring participants' acceptability of the program, as well as perceived needs for adjustments or improvements, through individual and focus group interviews conducted after program completion.

A secondary aim was to explore changes from baseline to end of the program in participants' self‐reported symptoms of ADHD, symptoms of stress, anxiety and depression, emotional regulation difficulties, and ADHD‐related quality of life and functioning using validated questionnaires.

## 2 Methods

The study conforms to better reporting of interventions: We report according to Intervention Description and Replication (TIDieR) guidelines [17] in the protocol and reporting for this study (Table S1).

### 2.1 Study Design

We conducted a mixed‐methods feasibility study. Quantitative data were collected using validated pre‐ and post‐intervention questionnaires, and qualitative data were collected through retrospective interviews following the intervention. The datasets were analysed separately, and the findings were integrated during interpretation.

### 2.2 Intervention

The purpose of AMOR is to provide participants with knowledge and practical tools to manage challenges associated with pregnancy and early motherhood while having ADHD. Psychoeducation and behavioural therapeutic approaches were used as the underlying frameworks. AMOR is based on an action‐oriented and practical approach, with a focus on strategies that can be implemented in everyday life. Emphasis is also placed on emotional regulation and coping skills to strengthen participants' ability to manage stress and impulses. In addition, the program seeks to support self‐understanding and self‐esteem by fostering insight into thoughts and behavioural patterns through guided reflection, group discussions, and peer support. Participants are introduced to techniques including breathing exercises, emotion regulation techniques, self‐compassion, and acceptance‐based strategies. Furthermore, the program includes practical methods for planning, prioritizing, and organizing tasks to support functioning and reduce everyday stress. Sharing experiences with other participants is a central component of the intervention with the aim to facilitate normalization, mutual support, and social connectedness.

AMOR consists of ten 90 min online group meetings held once a week at 10 a.m., complemented by ten podcast episodes (35–45 min each) covering a range of relevant topics (Figure 1).

**Figure 1:**
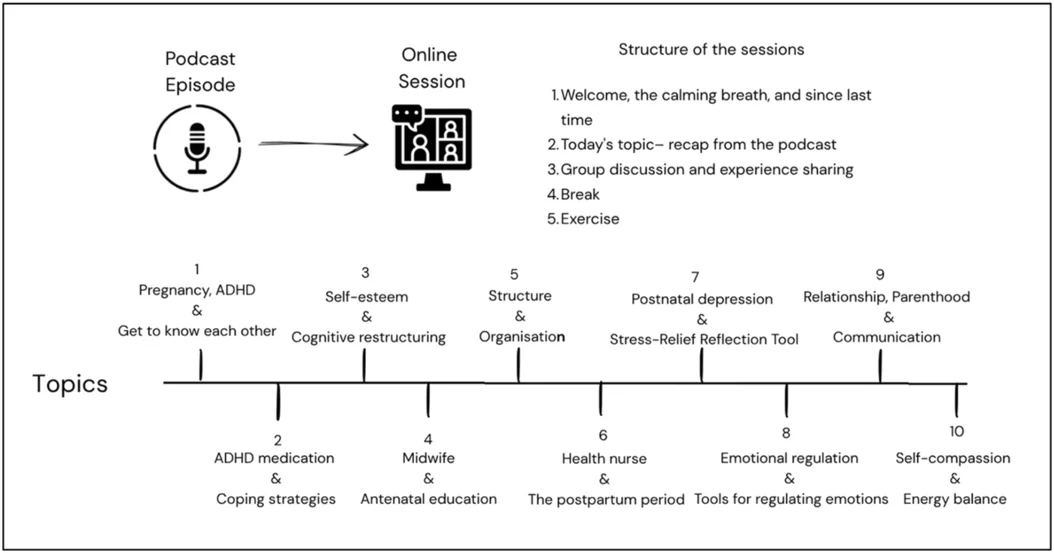
Overview of the AMOR program.

The podcast episodes are publicly available via any standard podcast application or platform. As part of the program, participants were encouraged to listen to the podcast episode corresponding to each session's topic prior to attending the session.

The online sessions were facilitated by a consultant from the Danish ADHD Association with a health professional background and experience in facilitating group sessions. Groups consisted of 8 to 12 participants. The same facilitator delivered all sessions across the three groups, following a standardized protocol specifying the session structure, topics and activities. Table S2 provides a detailed description of each podcast episode and group session.

### 2.3 Participants

Eligible participants where pregnant women with ADHD living in Denmark.

The *inclusion criteria* for the study were 1) age 18 years and above; 2) a self‐reported diagnosis of ADHD; 3) access to a computer and the internet, and 4) proficiency in spoken and written Danish.

The *exclusion criteria* were 1) other severe mental disorders, such as severe cognitive impairment, schizophrenia, psychosis, bipolar disorder or acute psychiatric conditions that required inpatient care; 2) self‐reported current alcohol or substance use disorder.

### 2.4 Procedure for Recruitment

We recruited through multiple sources: 1) the Danish ADHD Association's social media platforms, 2) hospital apps used to track the prenatal care journey, 3) Facebook ADHD groups, 4) family outpatient clinics for specialized support, and 5) midwives in standard maternity care services.

Participants self‐referred when each team were made available online in the period of December 2024 to March 2025 and enroled in the program through the Danish ADHD Association's website by providing their email address. Subsequently, they received an email through secure mail containing detailed information about the study. Approximately 1 week later, a consultant from the Danish ADHD Association contacted each individual by phone to conduct an eligibility screening. During the telephone eligibility screening, participants were asked to confirm that they had received an ADHD diagnosis and to report when they had been diagnosed. Eligibility was based on this reported diagnostic history; diagnoses were not independently verified through clinical assessment or medical records. All registered participants were contacted and offered placement in subsequent teams as available. Teams were manually closed once a sufficient number of participants was reached. Participants were assigned to one of three groups, with the first group starting in January, the second in February and the third in April 2025. Participants provided informed consent and completed baseline questionnaires between 15 January and 1 April 2025.

### 2.5 Data Collection

#### 2.5.1 Questionnaires

At baseline, prior to the first online session, participants completed online questionnaires distributed from the Research Electronic Data Capture (REDCap) about sociodemographic information and the following validated psychometric questionnaires; the Adult ADHD Self‐Rating Scale (ASRS) [18], the Generalized Anxiety Disorder assessment (GAD‐7) [19], the Edinburg Postnatal Depression scale (EPDS) [20], the Difficulties in Emotion Regulation Scale (DERS) [21], the Perceived Stress Scale (PSS) [22] and the Adult ADHD Quality of Life questionnaire (AAQoL) [23]. The ASRS and PSS recall periods were adapted to 14 days at both baseline and follow‐up to capture recent symptoms and experiences within a time frame appropriate to the 10‐week program. The same questionnaires were completed at follow‐up 1‐4 weeks after the last online session, along with the primary outcome questionnaires: the Client Satisfaction Questionnaire (CSQ‐8) [24] to assess satisfaction with the intervention, the Negative Effects Questionnaire (NEQ) [25] to assess potential adverse experiences associated with the program and two questions regarding credibility; specifically, whether participants would 1) recommend the program to others and 2) continue using the techniques from the program (Table S3). As the intervention facilitator did not have a therapeutic role, six NEQ items specifically related to the therapist relationship or therapist behaviour were not included (items 20, 21, 23, 25, 28, and 31). This resulted in a modified 26‐item version of the NEQ. Each item was rated on a 5‐point Likert scale ranging from 0 (“not at all”) to 4 (“extremely”), indicating the extent to which the reported negative effect affected the participant (impact).

#### 2.5.2 Interviews

During the final session of each program group, participants were invited to contact the first author (KM) if they wished to take part in an interview. Participation was voluntary, and no purposive selection was undertaken by the research team. Interview recruitment took place before completion of the follow‐up questionnaires. Five semi‐structured interviews were conducted 1–5 weeks after the final session of the program with a total of 12 participants. Three of the interviews were conducted individually and two were conducted as focus group interviews with 4 and 5 participants, respectively. All interviews lasted between 45 and 60 min.

The interview guide was developed with inspiration from Sekhon et. al.'s theoretical framework of acceptability of healthcare interventions [26]. Four constructs from the theory of acceptability were selected: (I) affective attitude, (II) burden, (III) opportunity costs, and (IV) perceived effectiveness. These constructs were chosen as they were applicable to participants' retrospective evaluations of the intervention and were used as themes in the interview guide and for coding the interviews. Specifically, they explored participants' motivation to engage in the program (affective attitude), their perceptions of the effort required to participate (burden), and the extent to which they experienced a need to give up potential benefits or opportunities to take part (opportunity costs). In addition, the interviews investigated participants' perceptions of the program's likelihood of achieving its intended outcomes (perceived effectiveness). Finally, we received insights into adjustments and improvements of the program (see the interview guide in Table S4).

### 2.6 Data Analysis

#### 2.6.1 Statistical Analysis

Descriptive statistics were used to summarize recruitment, program adherence, follow‐up retention, participant characteristics and changes in self‐reported outcomes, including ADHD‐, stress‐, anxiety‐, depression symptoms, emotion regulation difficulties, and ADHD‐related quality of life.

No numerical thresholds or formal progression criteria were pre‐specified for recruitment, retention, adherence, satisfaction or acceptability. These outcomes were evaluated descriptively, alongside qualitative accounts of participants' experiences, to inform further development and evaluation of the program.

Total scores range from 0 to 72 for the ASRS, 0–40 for the PSS‐10, 0–21 for the GAD‐7, 0–30 for the EPDS and 16–80 for the DERS‐16, with higher scores indicating greater symptom burden or difficulties. For the AAQoL, negatively directed items were reverse‐coded before summing all 29 items to obtain a raw total score ranging from 29 to 145, with higher scores indicating better ADHD‐related quality of life. AAQoL scores were not transformed to the standard 0–100 scale. Means and standard deviations were calculated for all continuous measures at baseline and follow‐up. Changes from baseline to follow‐up were examined using paired‐sample t‐tests to assess within‐subject differences between baseline and follow‐up. All analyses were conducted using Stata 18 BE (StataCorp LLC, College Station, TX, USA).

#### 2.6.2 Analytical Approach for the Interviews

The interviews were conducted online via Microsoft Teams by the first author (KM) and recorded. Transcripts were generated using the platform's automated transcription function and subsequently reviewed and corrected by KM. The interviews were analysed using the Framework Method, which provide a systematic and transparent approach to organising and interpreting qualitative data [27]. KM undertook the coding and analysis using NVivo and discussed the coding and interpretation with RKK. The analysis combined a deductive approach, guided by the four selected constructs from the Theoretical Framework of Acceptability [26], with an inductive exploration of participants' experiences. Subthemes were developed within all of the four main themes.

### 2.7 Ethical Considerations

All study procedures were approved by the Danish Regional Committees for Medical and Health Research Ethics (ID S‐20240074) and the Danish Data Protection Agency (Journal no 12.303), and the study was registered at ClinicalTrials. gov (S‐20240074, 5 November 2024). Informed consent was obtained from all participants prior to participation.

Participants with a score above 15 on the GAD or 18 on the EPDS at baseline were contacted to ensure appropriate support (*n* = 5).

## 3 Results

### 3.1 Recruitment, Retention and Adherence

Participants self‐referred and signed up for the program through the Danish ADHD Association's website, were each group reached full capacity within 24 h. Registration was closed when approximately 20 women had signed up for each group. Within 3 months, 57 pregnant women with ADHD signed up to take part in the study; 19 women were unable to be reached, and 7 were unable to participate due to work commitments or no longer being pregnant. A total of 32 participants were screened for eligibility and subsequently assigned to one of three groups including 9, 11 and 12 women, respectively. During the intervention period, five participants withdrew due to personal circumstances or work‐related commitments, and two participants did not complete the follow‐up assessments. Consequently, the final analytic sample consisted of 25 participants (Figure 2).

**Figure 2:**
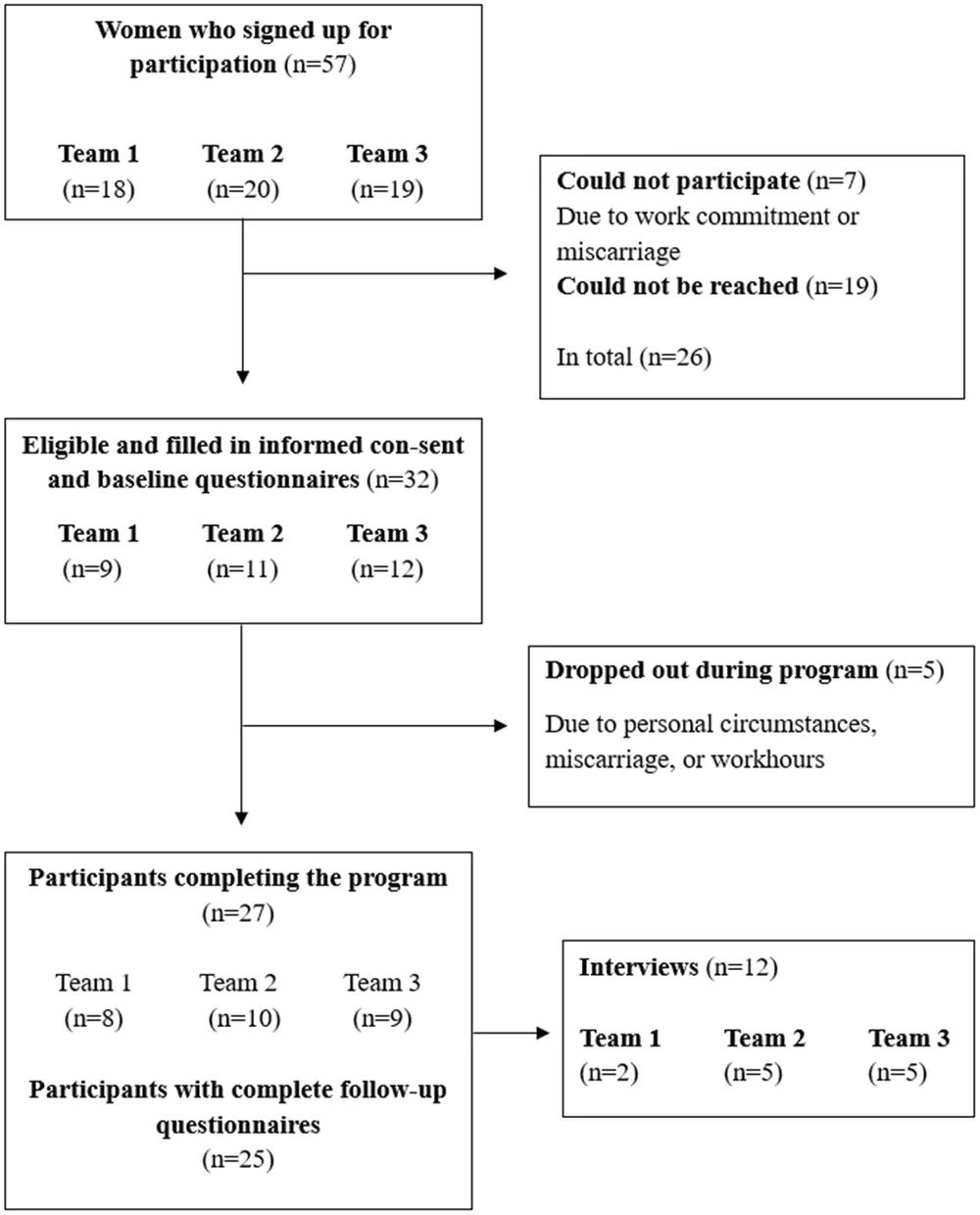
Flowchart of recruitment and numbers of participants in the AMOR program.

Program retention was assessed as completion of the intervention, with 27 of 32 participants (84.4%) completing the program. Follow‐up retention, defined as completion of the follow‐up questionnaires, was 92.6% (25/27). Adherence was measured by session attendance and ranged from 64% to 94%, with an average adherence rate of 80%.

### 3.2 Participant Characteristics

Of the participants, twenty were between the ages of 25‐34 years and 5 between 34 and 44 years. Three participants were diagnosed with ADHD before turning 18 years, 14 between the ages of 19‐29 years and eight were diagnosed in their thirties. Participants were at different stages of their pregnancy, 4 (16%) were in their first trimester, 16 (64%) in their second trimester, and 5 (20%) in their third trimester. Most participants were first time mothers, 21 (84%), and lived with their partner, 24 (96%). Eight (32%) were working full time when starting the program. Twenty‐two (88%) participants received standard maternity care and eight (32%) received additional care through a family outpatient clinic for specialized support. Eleven (44%) participants had other mental health diagnoses than ADHD, including depression, anxiety, eating disorder, OCD, autism or PTSD. Twelve (48%) participants were treated with ADHD medication and five (20%) with pharmacological treatment for depression or anxiety during pregnancy.

Twelve participants volunteered for interviews. Their age distribution was similar to that of the other participants. Eleven were first‐time mothers, and one had two children.

#### 3.2.1 Primary Quantitative Outcomes: Satisfaction, Credibility and Negative Effects

The Client Satisfaction Questionnaire (CSQ‐8) yielded a mean total score of 25.76 (SD: 5.58) out of a maximum score of 32. Of the participants, 22 (88%) reported being likely or very likely to recommend the program to others, while the rest were neutral. A total of 22 (88%) participants reported that they would continue using the techniques after the program.

The program was generally well tolerated and did not produce significant negative effects as measured with the NEQ. Of the 25 participants, 11 reported at least one negative effect attributed to the program. Most reported only one or two negative effects, while a few reported between four and six. Overall, reports of negative effects were infrequent and mild in severity. Endorsed items included ‘*The treatment did not suit me’* (*n* = 5, impact = “slightly”) and ‘*Old unpleasant memories were brought up’* (*n* = 4, impact = “slightly”). A few participants indicated ‘*I felt that the program (AMOR) did not have any effect’* (*n* = 4, impact = “slightly”) or that their ‘*expectations were not met’* (*n* = 3, impact = “slightly”). Isolated reports of increased anxiety (*n* = 1), hopelessness (n = 3), or reduced sense of competence (n = 1) were also noted, but these were rated as having low impact. No participants reported feeling stigmatized or afraid of others discovering their participation in the program.

#### 3.2.2 The Program's Acceptability

Themes and subthemes of the qualitative evaluation are presented in Table 1 along with example responses from participants.

**Table 1:** Themes and subthemes from individual‐ and focus‐groups interviews about acceptability of the AMOR program among pregnant women with ADHD (*n* = 12).

Themes	Sub‐themes	Example responses
Affective attitude	Need for knowledge and practical tools	‘*With having to manage without medication, I felt that at the very least I needed to get some tools to help me instead’ ‘I've just felt that I've been lacking knowledge—and especially targeted, relevant knowledge’*
	Worries of vulnerability and psychological challenges	‘*I've known that women can get postpartum depression. And since I myself, most likely due to my ADHD, have had several episodes of burnout with stress symptoms and depression throughout my life, I'm well aware that I'm in a risk group for depression. So, I think I've felt the need to take care of that’*
	Desire for community and support	‘*…also just be with others who were going through the same things as me. Because I quickly realized early in my pregnancy that what I was experiencing wasn't quite the same as what other pregnant women go through — I was really struggling’*
Burden	Reduced burden through online format	‘*I could manage it, even if I was having an off day or something — you can just log in and take part’ ‘…you could just take part from the couch, and it didn't require anything to show up — you could just log in’*
	Burden related to scheduling	‘*I wasn't on maternity leave yet when this program started, so I had to consider, like, okay, I actually need to step away for about an hour and a half in the middle of the day’*
Opportunity costs	No perceived sacrifice	‘*I was in the situation that I was on sick leave from my job when the program started, and then I was actually laid off—so I didn't have a job to attend to’*
	Sacrificing work	‘*Before I went on maternity leave, I actually found it a bit stressful because the sessions were during working hours, and I was already under pressure trying to fit it in. I could tell there were some days when I wasn't really fully present, because I actually should have been working at that time’*
Perceived effectiveness	Structure and strategies	‘*It helped to create structure around what was occupying my mind’* ‘*It encouraged concrete thinking about adjusting my own expectations’* ‘*It provided practical and concrete tools to handle challenges’*
	Increased self‐acceptance and self‐compassion	‘*The discussions about emotions and regulation gave me a lot—especially a sense of accepting where I'm at.’* ‘*I've become much kinder to myself and my inner dialogue.’* ‘*I'm more forgiving toward myself and the things that are difficult.’* ‘*I've shifted my focus—many of the worries have almost disappeared because I've started focusing more on myself.’*
	A sense of belonging and peer support	‘*It's nice to talk to someone you can relate to—to feel understood without having to explain everything’* ‘*I've often felt different or wrong… so it was amazing to meet all these competent, wonderful people.’* ‘*Seeing others with the same challenges made me think—if they can handle it, then I probably can too.’*
	Strengthened partner communication	‘*It inspired some really good conversations at home.’* ‘*I've had much better talks with my husband.’* ‘*I'm sure it will change how we parent together—there's now better planning and clearer expectations between us.’*
	Confidence in communicating with healthcare professionals	‘*It became easier to talk to health staff because I was more aware of what mattered to me’* ‘*It helped me realize that it's okay to mention my needs and ask for help.’* ‘*I feel better prepared to meet healthcare professionals and not mask as much.’*

#### 3.2.3 Affective Attitude

The subthemes regarding affective attitude reflected the various reasons for the women's motivation to participate in the program. Here, three sub‐themes emerged: *need for knowledge and practical tools, desire for community support*, and *worries about vulnerability and psychological challenges*.

Some women strongly desired information, guidance, and concrete strategies for managing pregnancy with ADHD, especially after discontinuing ADHD medication. Knowledge and tools were seen as essential for gaining control, preparing for motherhood, and preventing potential difficulties.

Another motivation to participate in the program was the women's sense of feeling different when sharing pregnancy‐related difficulties with friends. They described that strategies which had helped their friends during pregnancy were often ineffective for them, highlighting the value of sharing experiences and learning from other women with ADHD. Some participants also expressed concern about developing postpartum depression and their ability to cope with the challenges of motherhood while managing ADHD (without medication treatment).

#### 3.2.4 Burden

The subthemes related to *burden* encompassed both challenges with scheduling the program and the perceived *reduction of burden* through the online format. Some participants who were not yet on maternity leave found it difficult to explain to their workplaces why they needed to take time off during the day, as doing so would require disclosing their ADHD diagnosis. Others mentioned that it could be challenging to be present at the session when they felt they needed to be at work. All participants, however, expressed great relief that the sessions were held online, noting that for many, this format was what made participation possible.

#### 3.2.5 Opportunity Costs

The subthemes related to opportunity cost were described as either ‘no perceived sacrifice’ or ‘sacrificing work.’ The women who were on sick‐leave or had already begun their maternity leave felt that participation did not require giving up anything. They also appreciated the program's 10 a.m. scheduling, which they felt helped prevent delays later in the day. In contrast, women who were still working reported that participation required taking time off from work, and for those paid by the hour, this resulted in a loss of income.

#### 3.2.6 Perceived Effectiveness

Under the theme related to Perceived Effectiveness, five subthemes emerged. The participants felt they gained ‘*structure and strategies,’ ‘increased self‐acceptance and self‐compassion,’ ‘a sense of belonging and peer support.’ ‘strengthened partner communication’* and ‘*confidence in communicating with healthcare professionals.’*

Participants found the tools provided in the program to be concrete and useful for organizing thoughts, planning and navigating pregnancy with ADHD. They expressed that the guided exercises, reflections and group conversations helped them recognize and manage their emotions more effectively. For many, this led to greater perceived self‐acceptance and a kinder self‐view, often described as a sense of calm. One of the most frequently mentioned benefits was the sense of support and shared understanding among the women. Participants highly valued the opportunity to connect with peers during the sessions, and several expressed a wish for continued communication both between sessions and after the program concluded. Many also described improved communications with their partner. The program facilitated open dialogue about shared planning, emotional support and roles during pregnancy and the postpartum period.

Participants further noted that the reflection exercises helped them develop a shared language to articulate their emotions and needs. This made it easier to discuss expectations, selfcare, and emotional well‐being. For many, this translated into greater perceived confidence when communicating their needs, concerns and preferences with healthcare professionals, particularly regarding mental health, medication, and birth preparation. Some described this as a shift from feeling intimidated or dismissed to feeling empowered and legitimate in their experiences.

### 3.3 Adjustments and Improvements of the Program

Several adjustments and potential improvements were suggested by the participants. The women indicated an interest in *maintaining peer communication between sessions, extension of the program into the postpartum period, inclusion of partners*, and *modifications to the duration, composition, and overall structure of the sessions*.

A recurring theme was the wish to maintain contact with the other participants between sessions, with participants suggesting that this could be facilitated through a private online group. Many also proposed that the program should include a structured follow‐up component to support during the postpartum period. A strong wish was expressed to involve partners in at least one session. Participants felt that the information and discussions were highly relevant to their partners, and some reported that their partners were already engaging with the program by listening to the accompanying podcast series.

Participants further recommended adjustments to the structure and sequencing of the program. Women in the earlier stages of pregnancy suggested that the sessions with the midwife might be more beneficial if scheduled later in the program, allowing them to engage more meaningfully in childbirth related discussions. Several women proposed forming smaller discussion groups based on gestational stage, as they felt that reflections and needs sometimes differed depending on pregnancy stage. Nevertheless, many expressed appreciations for the diverse group composition, noting that it provided valuable insights and opportunities to learn from women at different stages of pregnancy. Finally, participants recommended extending each session by approximately 30 min to allow more time for reflection, discussion, and follow‐up exercises, as well as to ensure sufficient support for participants sharing sensitive experiences.

#### 3.3.1 Self‐Reported Mental Health

Table 2 presents exploratory changes in questionnaire scores from baseline to follow‐up among the 25 participants with complete data. Mean scores decreased for depressive symptoms, anxiety symptoms, difficulties in emotion regulation and ADHD symptoms, whereas perceived stress scores increased slightly. Raw AAQoL scores increased, indicating better self‐reported ADHD‐related quality of life.

**Table 2:** Secondary outcomes: changes in psychological and functional measures among participants before and after the intervention (*N* = 25).

Scale	Baseline Mean (SD)	Follow‐up Mean (SD)	Pre–post difference (follow‐up‐baseline) (95% CI)
Perceived Stress Scale (PSS)	22.08 (3.35)	22.60 (3.83)	+0.52 (+ 2.57; −1.53)
Edinburgh Postnatal Depression Scale (EPDS)	9.24 (4.97)	8.72 (4.54)	−0.52 (+ 1.06; −2.09)
Generalized Anxiety Disorder Scale (GAD‐7)	8.48 (4.87)	7.00 (3.36)	−1.48 (−0.03; −2.93)
Adult ADHD Quality of Life (AAQoL)*	88.08 (18.26)	96.60 (16.16)	+8.52 (+ 13.20; +3.84)
Difficulties in Emotion Regulation Scale (DERS‐16)	45.24 (14.80)	43.68 (14.53)	−1.56 (+ 2.22; −5.34)
Adult ADHD Self‐Report Scale (ASRS)	47.76 (8.74)	46.04 (11.85)	−1.72 (+ 1.99; −5.43)

* Higher scores indicate greater symptom severity on all measures except the Adult ADHD Quality of Life (AAQoL), for which higher scores reflect better quality of life.

## 4 Discussion

### 4.1 Summary of Findings

This mixed‐methods feasibility study evaluated the online group counselling program AMOR for pregnant women with ADHD in Denmark. Overall, the findings suggest that the program was feasible to deliver, well accepted by participants, and associated with high levels of satisfaction and perceived credibility. Recruitment feasibility was high, with all three study groups reaching full capacity within 24 h, indicating substantial unmet demand for ADHD‐adapted support during the pregnancy period. Among enroled participants, retention in both the program and follow‐up, as well as adherence was high, despite many still being employed and not yet on maternity leave.

The program appeared to be well tolerated, with few reported adverse experiences and no indications of severe negative effects associated with participation. Qualitative findings further supported the acceptability of the intervention, with participants describing the program as meaningful, supportive, and highly relevant to challenges experienced in relation to managing ADHD during pregnancy. In particular, participants emphasized the value of practical coping strategies, emotional support, normalization of experiences, and opportunities for peer connection with others facing similar difficulties.

Although the study was not designed or powered to evaluate effectiveness, small improvements were observed across several self‐reported psychological outcomes, particularly anxiety symptoms and ADHD‐related quality of life. Importantly, the qualitative findings suggested broader psychosocial participant‐perceived benefits that were not fully captured by symptom‐based questionnaires, including increased self‐acceptance, improved partner communication, greater confidence in interactions with healthcare professionals, and a stronger sense of preparedness for motherhood.

### 4.2 Feasibility and Acceptability of the Intervention

Several factors may explain the high feasibility and acceptability of the AMOR program. This likely reflects more than a general interest in ADHD‐related information and may indicate that existing perinatal services insufficiently address challenges commonly experienced by women with ADHD, including difficulties related to executive functioning, emotional regulation, stress management, and organization during pregnancy and preparation for motherhood [2]. Participants were highly motivated to engage in the intervention, reflected in the high adherence and in the qualitative findings describing a strong need for ADHD‐specific knowledge, practical coping tools, emotional support, and opportunities to connect with other women facing similar challenges during pregnancy. Many participants also expressed concerns about psychological vulnerability, stress, and postpartum depression, particularly in relation to managing pregnancy and motherhood with ADHD symptoms and, for some, without pharmacological treatment. The qualitative findings suggested that these needs were meaningfully addressed through the intervention.

The online format appeared to be central to participation and adherence. Participants consistently described the online format as essential for their ability to attend, as it reduced practical burdens related to transportation and the time required to attend in person. In contrast to previous online interventions for pregnant women that primarily relied on self‐guided app‐based communication and reported lower adherence [28], AMOR combined accessibility with live interaction and peer support. The group‐based format appeared important for acceptability, with participants describing that they felt acknowledged and understood in the group. Many emphasized that sharing experiences with peers who faced similar challenges created a sense of normalization and belonging, which may have contributed to engagement and the positive group climate. Previous research suggest that group climate and perceived social support are important factors for participants' outcomes in psychosocial interventions [29].

Finally, participant characteristics may also have contributed to motivation and engagement. Almost all participants were first‐time mothers, and one third were diagnosed after the age of 30 years, suggesting that some women may have been navigating both a relatively recent understanding of their ADHD and the transition to motherhood simultaneously.

### 4.3 Integration of Qualitative and Quantitative Findings

Although the quantitative changes observed in behavioural and psychological symptoms and functioning were small, the qualitative findings suggested broader psychosocial changes that were not captured by the symptom‐based questionnaires. Experiences described by participants were primarily related to greater perceived self‐understanding, confidence, emotional coping, normalization of experiences, and interpersonal functioning rather than marked symptom reduction. This may be particularly relevant in a feasibility study of a supportive psychosocial intervention during pregnancy, where meaningful changes may emerge initially through increased coping capacity, self‐acceptance, and perceived preparedness rather than through reductions in symptom severity over a relatively short intervention period.

The qualitative findings provide context for the small improvements in the quantitative outcomes. Participants described increased self‐acceptance and self‐compassion, with many reporting a shift away from self‐criticism and worry towards greater acceptance of their own needs and limitations, which appeared to foster a greater sense of calmness and self‐care. These experiences may be consistent with the small reduction observed in anxiety symptoms. Likewise, participants did not describe their ADHD symptoms as disappearing, but rather as becoming more manageable through concrete coping strategies, planning tools, and adjusted expectations. This may help explain why improvements were more apparent in ADHD‐related quality of life than in ADHD symptom severity, although the relationship between perceived benefits and changes in scores was not established.

Beyond symptom change, the qualitative findings point to processes that may be central to perinatal adaptation among women with ADHD. Participants described feeling normalized, understood, and legitimate in their experiences, rather than feeling different or inadequate compared with pregnant women without ADHD. This was particularly evident in accounts of peer support, reduced self‐criticism, increased self‐compassion, and feeling better able to express needs without masking difficulties. These processes may be clinically important because they suggest a shift from managing ADHD primarily as an individual deficit toward understanding ADHD‐related challenges as legitimate needs that can be anticipated, communicated, and supported during pregnancy and early motherhood [2, 5].

Several qualitative themes may further represent potential mechanisms through which psychosocial interventions could influence longer‐term maternal well‐being during pregnancy and early motherhood. Participants described *strengthened partner communication*, and increased *confidence in communicating with healthcare professionals*. Improved partner communication, including shared planning and clearer mutual expectations may help contextualize the improvement in anxiety symptoms and ADHD‐related quality of life. Notably, confidence in interaction with healthcare professionals was not described as a primary motivation for participation but emerged as an important perceived benefit of the program. Participants explained that increased insights into their own needs and emotional responses made it easier to ask for help and communicate preferences related to pregnancy, mental health, and preparation for motherhood. A mixed‐methods systematic review from 2022 underpins that recognising appropriate and inappropriate support from health professionals are important factors for women's level of depression and anxiety symptoms in the perinatal period [30].

The qualitative findings also suggested potential changes in constructs related to maternal self‐efficacy. Increased perceived self‐acceptance, self‐compassion and confidence in managing everyday challenges is closely aligned with the theory of self‐efficacy, i.e. a person's belief in their ability to successfully carry out a specific task or handle a particular situation [31]. A recent study found that high maternal self‐efficacy was associated with high maternal role competence and fewer postpartum depression symptoms [32]. This highlights the importance of individuals’ perceptions of their own maternal skills. Future research evaluating the AMOR program may therefore benefit from including maternal self‐efficacy to better capture potential psychosocial changes that may not be adequately reflected in traditional symptom‐based measures alone.

### 4.4 Strengths and Limitations

A major strength of the study was the mixed‐methods design, which enabled exploration of potential underlying mechanisms that may explain participants' perceived benefits of the program. Another important strength was the high response rate and the large number of participants in qualitative interviews, providing rich and in‐depth data on participant experiences.

Some important limitations of this study should be acknowledged. First, the absence of pre‐specified feasibility thresholds limits conclusions about whether recruitment, retention, adherence, satisfaction and acceptability were sufficient to support progression to a definitive trial. The findings should therefore inform future study planning rather than be interpreted as demonstrating that formal progression criteria were met. In addition, although the program was standardized and delivered by the same facilitator across all groups, fidelity was not formally measured, limiting assessment of how consistently the planned content was delivered. Also, ADHD diagnosis was self‐reported and not verified clinically, however, eligibility screening included explicit questions about whether participants had received an ADHD diagnosis and when they had been diagnosed. This provided information about their diagnostic history, although diagnostic accuracy could not be confirmed. In addition, participant outcomes were assessed at different stages of pregnancy, and physiological as well as psychological changes across pregnancy may have influenced participants' responses. Medication use was assessed only at baseline, so any subsequent initiation, discontinuation or dose changes were unknown and may also have contributed to the observed pre–post changes. Furthermore, self‐referral may have resulted in a sample with greater motivation, engagement and help‐seeking behavior than the broader population of pregnant women with ADHD. The online format and requirement for internet access may also have favoured women with greater digital access or confidence using online services. These selection factors may limit generalizability to women who are less inclined to seek support or face greater barriers to online participation. Also, participants may have been inclined to provide positive evaluations because interviews were conducted shortly after program completion. Finally, the quantitative analyses were underpowered and without a control group, observed changes may reflect natural adaptation during pregnancy.

## 5 Conclusion

The online group program AMOR appeared to be a feasible, acceptable, and well‐tolerated intervention for pregnant women with ADHD. Its acceptability was reflected in participants' descriptions of the program as meaningful, supportive, and highly relevant to the challenges associated with managing ADHD during pregnancy. Although quantitative improvements in symptom‐based measures of ADHD, anxiety, and depression were small, qualitative findings suggested meaningful psychosocial benefits. The findings further suggest that outcomes related to maternal self‐efficacy, perceived social support, and healthcare communication may be important targets for future evaluations.

## Conflicts of Interest

The authors declare no conflicts of interest.

## Supporting information


Supporting File 1

Supporting File 2

Supporting File 3

Supporting File 4

## Data Availability

De‐identified data supporting the findings of this study may be made available from the corresponding author upon reasonable request, subject to applicable data protection requirements.
