# Supplementary material for: A Mixed‐Method Feasibility Study of an Online Group Program for Pregnant Women With ADHD
Source: J Eval Clin Pract. 2026 Nov 15;32(8):e70755. doi: 10.1111/jep.70755 (PMC13770502; doi:10.1111/jep.70755)
Supplement: Supplementary file 2 [file JEP-32-0-s001.docx]

| **Affective Attitude**  *How the participant feels about the intervention* | - "Where did you first hear about AMOR/the program?" - "What was your motivation or reason for enrolling in the program?"   - (Did the timing of your diagnosis, ADHD symptoms, stopping medication, or encouragement from others play a role?) - "Were any of you unsure about whether you wanted to participate?"   - If yes: "What made you hesitant?"   If no “why?” |
| --- | --- |
| **Burden**  *The perceived amount of effort required to participate in the intervention* | - "Has participating in the program been demanding?"   - If yes: "What has made it demanding?" (e.g., external circumstances, the program exercises, listening to the podcasts, or something else?) - "Were there any particular sessions that you found especially demanding?"   - If yes: "Was this due to the content, the exercises, or something else?" - "Were there any exercises that you found difficult to complete?" - "What do you think about the length of the program (10 sessions over 10 weeks)? Would you have preferred it to be longer, shorter, or was the duration appropriate?" - "What do you think about the timing of the sessions (morning)? How did it fit with work or family responsibilities, if applicable?"   "If you could change anything about the program, what would it be?" |
| **Opportunity Costs**  *The benefits, opportunities, or valued activities that participants may have had to give up to participate* | - "Are there any specific activities or opportunities that you feel you have missed out on while participating in the intervention?" - "Have you changed any of your priorities or goals in order to take part in this intervention?" - "Is there anyone or anything you feel you have neglected because of your participation in the intervention (e.g., family, work, or leisure activities)?" |
| **Perceived Effectiveness**  *The extent to which the intervention is perceived as achieving its intended purpose* | - "How was your experience of participating online?"   - "Would you have preferred to attend in person?" - "Overall, what did you think about the program?" - "What did you like about the program?" - "Did you feel that the podcasts and group sessions addressed your specific needs as a pregnant woman with ADHD?" - "Did the program help you do things differently in your everyday life?"   - "How did it help you?" - "Did the group sessions provide anything that the podcasts alone could not?" - "Was there anything about the program that you did not like?" - "Has the counselling program changed the way you think about being diagnosed with ADHD?"   - "Do you see your challenges or strengths differently now?"   - "How?" |
