# Supplementary material for: A Mixed‐Method Feasibility Study of an Online Group Program for Pregnant Women With ADHD
Source: J Eval Clin Pract. 2026 Nov 15;32(8):e70755. doi: 10.1111/jep.70755 (PMC13770502; doi:10.1111/jep.70755)
Supplement: Supplementary file 3 [file JEP-32-0-s002.docx]

| **Podcast 1**  26 min.  *Topic: Pregnancy & ADHD Host:* Consultant from the Danish ADHD association Attending the podcast: Psychiatrist specializing in ADHD   - What is ADHD? - How hormones affect ADHD symptoms - Medication and how it can be monitored during menstrual cycle - How hormones can affect ADHD during pregnancy, after birth - How breastfeeding can have an impact on ADHD symptoms - Postpartum depression and ADHD - Sleep deprivation - Heritability of ADHD - Medication and postpartum depression | **Meeting 1**  10 min Welcome, introduction of the instructor and course 15 min Participants introduce themselves 15 min Get-to-know-each-other quiz  15 min Break 10 min Today's topic – recap from the podcast 20 min Group discussion and experience sharing 5 min Recap and final comments |
| --- | --- |
| **Podcast 2** 37 min.  *Topic: Medication & ADHD Host:* Consultant from the Danish ADHD association Attending the podcast: Researcher and General practitioner   - The different ADHD medication types: stimulant and non-stimulant - Pregnancy and medication - The risk-risk trade off between potential adverse effect on the child and maternal mental health - The conversation with doctor before deciding whether to stop or continue ADHD medication when trying to get pregnant - The risk of postpartum depression | **Meeting 2** Welcome and since last time Today's topic – recap from the podcast Group discussion and experience sharing Break Coping as a process  Recap and final comments |
| **Podcast 3** *Topic: Self-esteem & ADHD* 43 min.  *Host:* Consultant from the Danish ADHD association  Attending the podcast: ADHD specialist and mother with ADHD   - The definition of self-esteem and how it is different from self-confidence - Lower self-esteem related to ADHD - Setting boundaries and how boundaries   can be difficult to identify and why   - Being aware of thought patterns - Working with affirmation | **Meeting 3**  Welcome, the calming breath, and since last time Today's theme – recap from the podcast Group discussion and experience sharing Break Exercise - Changing negative thoughts and behaviours patterns. Recap and final comments |
| **Podcast 4** *Topic: Advice from the midwife*  43 min.  *Host:* Consultant from the Danish ADHD association  Attending the podcast: Midwife.   - Talking about ADHD with the midwife - Different care options for women with ADHD during pregnancy - The emotional system can feel different under pregnancy - How the birth can appear stressful and how it feels afterwards - How being aware of your body impacts birth - MUMflow, breathing exercise – focus on the body | **Meeting 4**  Welcome and since last time Today's theme – recap from the podcast Group discussion and experience sharing Break Exercise – create a birth plan Recap and closing |
| **Podcast 5** *Topic: Structure and organizing*  44 min.  *Host:* Consultant from the Danish ADHD association  Attending the podcast: ADHD specialist and pregnant woman with ADHD   - How structure gets more important after kids - Why recommend structure for people having difficulties with structuring? - Finding a system that works - Prioritize - Importance of breaks - It takes time to get structured | **Meeting 5**  Welcome, the calming breath, and since last time Today's theme – recap from the podcast Group discussion and experience Break Group exercise – baby’s first year, what needs to be done and are there any deadlines? Recap and final comments |
| **Podcast 6** *Topic: Advice from the health nurse*  33 min.  *Host:* Consultant from the Danish ADHD association  Attending the podcast: Health nurse/health visitor  - the role of the health nurse   - How to get a good start with a newborn - Worries about changing structures after birth - Breastfeeding  - How can the partner help - How to take breaks and knowing it’s okay not to be perfect all the time - Openness about what is on your mind to the health nurse or in a maternal postpartum group | **Meeting 6**  Welcome, the calming breath, and since last time Today's theme– recap from the podcast Group discussion and experience Break Group exercise – Is it important to you? *Questions for participants to reflect on:*   - *Is it important to you that the health visitor knows about your ADHD diagnosis?* - *Is it important to you that your relationship with your health visitor is so trusting that you can share everything with her?* - *Is it important to you how you and your home appear when your health visitor comes for a visit?* - *Is there anything else that is important for you that your health visitor should know about you, be aware of, or help you with?*   Recap and final comments |
| **Podcast 7** *Topic: Postpartum depression and ADHD* 37 min.  *Host:* Consultant from the Danish ADHD association  Attending the podcast: Researcher and mother with ADHD   - What is postpartum depression - What research shows about ADHD and pospartum depression - Personal story about postpartum depression and ADHD | **Meeting 7**  Welcome, the calming breath, and since last time Today's theme – recap from the podcast Group discussion and experience Break Group exercise – Mind map your network  5 minutes alone and 10 minutes in smaller groups – group recap   - *What is important for keeping your stress level down?* - *Who can help you with what?* - *The things you don’t ask for help with: Do you have any good strategies that you can use to make those things happen?*   Recap and final comments |
| **Podcast 8** *Topic: Emotion regulation and ADHD* 35 min. *Host:* Consultant from the Danish ADHD association Attending the podcast: ADHD specialist and mother with ADHD   - What is emotional regulation? - How does emotions connect to behaviour - Personal story about how difficulties with emotional regulation shows in everyday life - Why is it difficult for people with ADHD to regulate emotions? - Why can it be a challenge not to be able to regulate own emotions? - Pregnancy, hormones and how it can contribute to emotion regulation - What can you do to get better at regulating emotion? - Examples on strategies to help regulate emotions | **Meeting 8**  Welcome, the calming breath, and since last time Today's theme – recap from the podcast Group discussion and experience Break Group exercise – Emotion regulation  There are many different strategies for emotional regulation – it takes practice to know what works for you.  *Use your body* - followed by example *Use your breathing* – followed by example *Create a mantra* – followed by example  *Withdraw from the situation*  Recap and final comments |
| **Podcast 9** *Topic: Relationship, parenthood and ADHD* 44 min. *Host:* Consultant from the Danish ADHD association Attending the podcast: Specialist in ADHD and a couple with two children where the mother is diagnosed with ADHD.   - How does the partner notice ADHD in everyday life - How does ADHD affect the relationship? - The “project manager” at home – how is organising at home structured? - How the partner can misinterpret ADHD symptoms - Dynamics in the relationship - How emotion regulation can impact the relationship - Transition into parenthood | **Meeting 9**  Welcome, the calming breath, and since last time Today's theme – recap from the podcast Group discussion and experience Break Group exercise – Include your partner 5 minutes alone – 10 minutes in groups – recap  *Think about what you would write in a letter/speech to your partner if you had to describe things that are important for you to express – both for your relationship and your parenting* Recap and final comments |
| **Podcast 10** *Topic: Self-compassion and energy balance*  41 min.  *Host:* Consultant from the Danish ADHD association  Attending the podcast: ADHD specialist and mother with ADHD   - How do people with ADHD experience energy balance? - Personal story about the struggle of finding the right energy balance - Why is it difficult to balance energy with ADHD? - How the energy balance impact emotion regulation - Energy optimizing vs balancing energy - Find your breaks - Prioritizing and thoughts about how to structure maternity leave | **Meeting 10**  Welcome, the calming breath, and since last time Today's theme – recap from the podcast Group discussion and experience Break Group exercise – Prioritizing and getting better to saying no - *Identify what is meaningful for you to say yes to, and explore the feelings associated with saying no.*  Recap and final comments |
