# Supplementary material for: A Mixed‐Method Feasibility Study of an Online Group Program for Pregnant Women With ADHD
Source: J Eval Clin Pract. 2026 Nov 15;32(8):e70755. doi: 10.1111/jep.70755 (PMC13770502; doi:10.1111/jep.70755)
Supplement: Supplementary file 4 [file JEP-32-0-s003.docx]

| **Supplementary Table 3. Overview of questionnaires and assessment** | | | | | | | |
| --- | --- | --- | --- | --- | --- | --- | --- |
| **Questionnaires/assessment** | **Assessed at:** | | | **Items (n)** | **Score range** | **Adjustments** | **Changes from original** |
| **Primary outcomes** | | | | | | | |
|  | **Baseline** | **Follow-up** | |  |  |  |  |
| The Client Satisfaction Questionnaire (CSQ-8) (1) |  |  | | 8 | 8–32 |  |  |
| Two questions assessed participants’ willingness to recommend the program to others and their intention to continue using the techniques from the program. |  |  | |  |  | “How likely are you to recommend the program to a friend with similar problems?”  “How likely are you to continue using the techniques from the program?” |  |
| The Negative Effects Questionnaire (NEQ) (2) |  |  | | 26 | 0-104 | In the questionnaire the word “treatment” was clarified as referring to the program. | 26 items were used (original is 32): Question 20, 21, 23, 25, 28 and 31 were removed. The questions related specifically to the therapist. Since the facilitator did not have a therapeutic function, these questions were excluded. |
| Adherence | Attendance was noted at the beginning of each meeting | | | 10 meetings | 0-100% |  |  |
| **Secondary outcomes** | | | | | | | |
| The Adult ADHD Self-Rating Scale (ASRS) (3) |  | |  | 18 | 0-72 |  | The original questionnaire refers to a period of 6 months. The follow up questionnaire was changed to14 days. |
| The Perceived Stress Scale (PSS) (4) |  | |  | 10 | 0-40 |  | The original questionnaire refers to a period of 6 months. The follow up questionnaire was changed to14 days. |
| The Generalized Anxiety Disorder assessment (GAD7) (5) |  | |  | 7 | 0-21 |  |  |
| The Edinburg Postnatal Depression Scale (EPDS) (6) |  | |  | 10 | 0-30 |  |  |
| The Difficulties in Emotion Regulation Scale (DERS-16) (7) |  | |  | 16 | 16-80 |  |  |
| The Adult ADHD Quality of Life Scale (AAQoL) (8) |  | |  | 29 | 29-145 |  |  |

**References**

1.	Attkisson CC, Zwick R. Client Satisfaction Questionnaire-8 [Internet]. 2016 [cited 2026 May 7]. Available from: https://doi.apa.org/doi/10.1037/t50119-000 doi:10.1037/t50119-000

2.	Rozental A, Kottorp A, Forsström D, Månsson K, Boettcher J, Andersson G, et al. The Negative Effects Questionnaire: psychometric properties of an instrument for assessing negative effects in psychological treatments. Behav Cogn Psychother. 2019 Sep;47(5):559–72. doi:10.1017/S1352465819000018

3.	Van De Glind G, Van Den Brink W, Koeter MWJ, Carpentier PJ, Van Emmerik-van Oortmerssen K, Kaye S, et al. Validity of the Adult ADHD Self-Report Scale (ASRS) as a screener for adult ADHD in treatment seeking substance use disorder patients. Drug and Alcohol Dependence. 2013 Oct;132(3):587–96. doi:10.1016/j.drugalcdep.2013.04.010

4.	Eskildsen A, Dalgaard VL, Nielsen KJ, Andersen JH, Zachariae R, Olsen LR, et al. Cross-cultural adaptation and validation of the Danish consensus version of the 10-item Perceived Stress Scale. Scand J Work Environ Health. 2015 Sep;41(5):486–90. doi:10.5271/sjweh.3510

5.	Spitzer RL, Kroenke K, Williams JBW, Löwe B. A Brief Measure for Assessing Generalized Anxiety Disorder: The GAD-7. Arch Intern Med. 2006 May 22;166(10):1092. doi:10.1001/archinte.166.10.1092

6.	Smith-Nielsen J, Matthey S, Lange T, Væver MS. Validation of the Edinburgh Postnatal Depression Scale against both DSM-5 and ICD-10 diagnostic criteria for depression. BMC Psychiatry. 2018 Dec 20;18(1):393. doi:10.1186/s12888-018-1965-7 PubMed PMID: 30572867; PubMed Central PMCID: PMC6302501.

7.	Bjureberg J, Ljótsson B, Tull MT, Hedman E, Sahlin H, Lundh LG, et al. Development and Validation of a Brief Version of the Difficulties in Emotion Regulation Scale: The DERS-16. J Psychopathol Behav Assess. 2016 Jun;38(2):284–96. doi:10.1007/s10862-015-9514-x

8.	Brod M, Johnston J, Able S, Swindle R. Validation of the Adult Attention-Deficit/Hyperactivity Disorder Quality-of-Life Scale (AAQoL): A Disease-Specific Quality-of-Life Measure. Qual Life Res. 2006 Feb;15(1):117–29. doi:10.1007/s11136-005-8325-z
